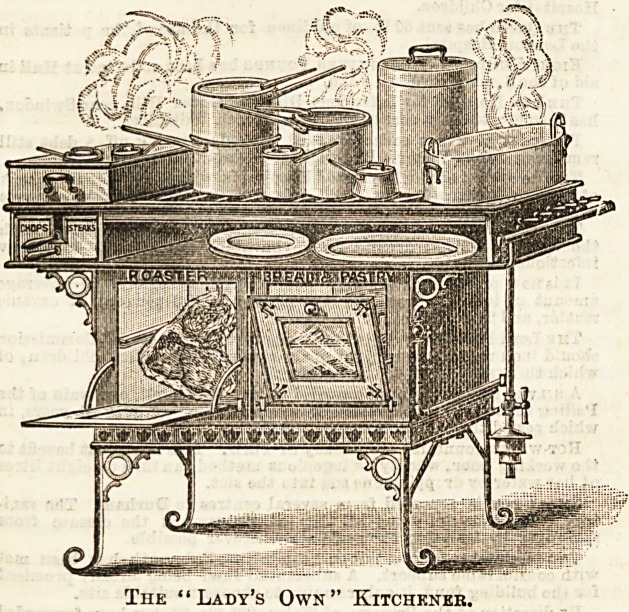# Cooking by Gas. III

**Published:** 1893-01-21

**Authors:** 


### PRACTICAL DEPARTMENTS.

### COOKING BY GAS.—III.

PRIVATE HOUSEHOLDS.

The requirements of small households differ much more than those of large establishments. In large establishments, other than hotels and restaurants, it is merely a question of larger or smaller apparatus of the same description. Small households require practically totally different contrivances for each individual kitchen in proportion to the amount of cooking required. This the manufacturers have realised, and there is rather an *embarras de richesse* than a dearth of suitable contrivance of every description. In fact, we have experienced a good deal of perplexity in choosing subjects for illustration, so admirable are the productions of many makers. Messrs. Sugg are especially successful in supplying, and even in forestalling, the wants of the household, and the not less important individual, and we are indebted to them, and through them to Messrs. Cassell, for our illustrations, which in cases of this kind are far more descriptive than words. It will be readily understood that whilst in large establishments, employing a numerous staff in the kitchen, separate apparatus for each branch of cooking is desirable, in private households compactness is the essential feature. We have chosen two kitcheners, illustrated below, as being especially commendable for an average kitchen, as possessing all the qualifications requisite. Their compactness strikes one at a glance. They are economical in the consumption of gas, and easy to keep clean, the ovens and the plate above both being enamelled white. This fact alone will unfailingly recommend them to the orderly housewife. Either of these kitcheners is of sufficient size to cook a dinner for twelve persons.

"The Westminster" measures but 30 inches in the widest part, 26 inches in the deepest, and stands 35 inches high. Within this small space roasting, grilling, baking, and boiling can be effectively accomplished, whilst space is also afforded for the heating of plates.

"THE WESTMINSTER" KITCHENER.

The lady's own kitchener, our second illustration, is of more elegant design, and there is also more space for boiling, a distinct advantage where room can be afforded.

The "Charing Cross" is an excellent kitchener on a larger scale than either of the two we have mentioned, and the oven of this is provided with a glass panel to the door, which is an extra convenience, but may be an additional source of expense, unless care is exercised. These kitcheners, and all Messrs. Sugg's kitchen fittings are made on the interchangeable system, which allows any broken part to be replaced without difficulty or delay. This is no small advantage, as it removes the disagreeableness of being at the mercy of an unskilled ironmonger.

Most of the kitcheners can be had with luminous or atmospheric burners, but the luminous burners are generally preferred. All ladies should insist, as a matter of safety, on having flash lights attached to their kitcheners.

We are prepared for one grave objection to the use of gas apparatus in private households, which has possibly appeared insuperable to many. We refer to the absence of boiler in connection with gas kitcheners. This difficulty struck us at the onset, and inquiries failed to bring forth any satisfactory solution until we met with an excellent contrivance invented by Professor Vernon Harcourt. This apparatus is a boiling steam Therma, which is cylindrical in form, and can be had in different sizes. It is intended that it should be fixed to the scullery sink, that the water supply may come from thence, and that hot water may be conveniently situated. The heating is by gas from below; and after the water is once heated a very small flame will suffice to keep it at a sufficiently high temperature. A good supply of hot water is so essential that it is a mistake to endeavour to do without it, and the kettle is no substitute for a boiler. The Therma overcomes the difficulty and will serve to render the kitchen service convenient and complete. There is one addition which we feel sure will add to the comfort of every kitchen, which, were we ourselves occupied in cooking, we should consider even an essential where gas is used. That is a small table, covered with a thin metal plating at the top, on to which pans em ty or required for use again might be placed. The

THE "LADY'S OWN" KITCHENER.

convenience of such an arrangement, where the actual cooking space is so small as in these gas kitcheners, will be apparent at once to all those who have practical knowledge of cooking and kitchen arrangements. With the last addition, we feel that little can be said against the convenience of applying gas to kitchen use.

## Figures and Tables

**Figure f1:**
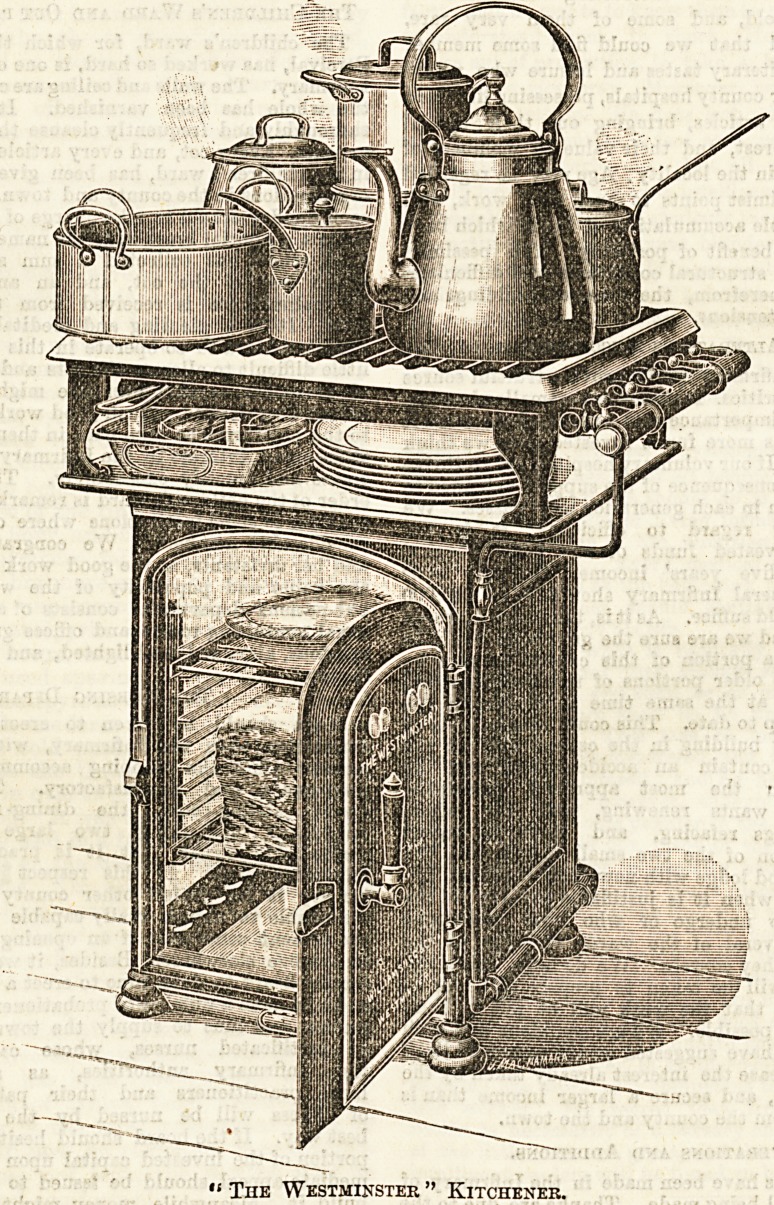


**Figure f2:**